# Intense pulsed light annealing of solution-based indium–gallium–zinc–oxide semiconductors with printed Ag source and drain electrodes for bottom gate thin film transistors

**DOI:** 10.1038/s41598-024-52096-2

**Published:** 2024-01-18

**Authors:** Chang-Jin Moon, Jong-Whi Park, Yong-Rae Jang, Hak-Sung Kim

**Affiliations:** 1 Department of Mechanical Engineering, Hanyang University, Haengdang-Dong, Seongdong-gu, Seoul, 133-791 Republic of Korea; 2 Institute of Nano Science and Technology, Hanyang University, Seoul, 133-791 Republic of Korea

**Keywords:** Electrical and electronic engineering, Nanoscale materials

## Abstract

In this study, an intense pulsed light (IPL) annealing process for a printed multi-layered indium–gallium–zinc–oxide (IGZO) and silver (Ag) electrode structure was developed for a high performance all-printed inorganic thin film transistor (TFT). Through a solution process using IGZO precursor and Ag ink, the bottom gate structure TFT was fabricated. The spin coating method was used to form the IGZO semiconductor layer on a heavily-doped silicon wafer covered with thermally grown silicon dioxide. The annealing process of the IGZO layer utilized an optimized IPL irradiation process. The Ag inks were printed on the IGZO layer by screen printing to form the source and drain (S/D) pattern. This S/D pattern was dried by near infrared radiation (NIR) and the dried S/D pattern was sintered with intense pulsed light by varying the irradiation energy. The performances of the all-printed TFT such as the field effect mobility and on–off ratio electrical transfer properties were measured by a parameter analyzer. The interfacial analysis including the contact resistance and cross-sectional microstructure analysis is essential because diffusion phenomenon can occur during the annealing and sintering process. Consequently, this TFT device showed noteworthy performance (field effect mobility: 7.96 cm^2^/V s, on/off ratio: 10^7^). This is similar performance compared to a conventional TFT, which is expected to open a new path in the printed metal oxide-based TFT field.

## Introduction

A flexible and foldable display is a next-generation display, which also required high-quality visualization^1–3^. With this trend, the semiconductor materials of thin film transistors (TFTs) such as Si-based compounds have been replaced by oxide semiconductors such as indium–gallium–zinc–oxide (IGZO)^1–5^. IGZO has received much attention as a channel layer of next generation TFTs due to its optical transparency, high electron mobility, atmospheric stability, and mechanical flexibility^6–8^. Generally, photolithography has been used to fabricate IGZO in a TFT, which involves complicated processes of deposition, exposure, and etching processes, resulting in high process costs. To improve the process efficiency and reduce the manufacturing cost, a solution-processed IGZO combined with a printing technique was recently developed, which can form a semiconductor film without photolithography at room temperature and atmospheric pressure^9–12^. The performance and process efficiency of solution-processed IGZO has been improved through the development of annealing processes and breakthrough in materials and is comparable to that of deposited IGZO^9,10,13–17^. However, a deposition process has still been employed to form materials such as electrodes and dielectrics as part of the TFT fabrication process. As a result, the efficiency of the total process has not yet been improved. In the past decade, printed electronics technology has been rapidly developed, utilizing printing technology to form electrodes with various nano-ink materials without a photolithography process^18–21^. Through printed electronics technology, it has become possible to fabricate electrodes by a simple process of printing and sintering, and various printed type devices have been introduced in recent years^18,22–26^. Various metal materials have been used in IGZO-based TFTs and the correlation between the TFT performance and metal electrode was evaluated^27–31^. In the case of molybdenum and titanium, it was possible to improve the TFT performance due to their low contact resistance with IGZO, but it has hardly been used as an ink material due to difficulty in handling the nanoparticles because of their oxidation phenomena in atmospheric conditions. On the other hand, silver (Ag) is the most advanced electrode material in printed electronics because of its oxidation resistance and chemical stability^32,33^. Y. Ueoka reported that Ag could be used as an electrode of IGZO-based TFTs in a specific driving range^29^. However, since the sintering of the printed Ag electrode requires the application of heat to the Ag electrode as well as IGZO layer, several additives from the Ag ink deteriorate the TFT performance as the IGZO channel is contaminated by them^34,35^. In order to reduce these interfacial reactions, an interlayer between the IGZO channel and electrode was employed^36^. However, since the sintering process of the interlayer requires not only a heat process but also a long process time, it is inefficient. In order for printed electronics to be used more effectively in TFT fabrication, several innovative changes are needed in the sintering or annealing process. To address the long sintering time, intense pulsed light (IPL) sintering technology was developed to selectively heat nanomaterials such as the metal electrode and thin film in a short time under ambient conditions at room temperature^17,25,37–45^. Using IPL sintering technology, oxide semiconductor layers as well as metal electrodes could be annealed effectively in a short process time. However, to the best of our knowledge, an IPL-sintered metal electrode was not successfully used yet on IGZO for TFTs while the deposited Al electrode was used on an IPL-annealed IGZO layer^17,25^. This might be because most previous studies have focused only on the annealing of each single layer of IGZO or electrode. Simultaneous annealing and sintering of the semiconductor layers and metal electrode with an IPL has not been studied yet, which may provide an innovative technique for the TFT manufacturing process.

Therefore, in this study, an IPL annealing process of printed multi layers composed of printed IGZO and a printed Ag electrode was developed combined with deep-UV (DUV) and near-infrared (NIR) drying for high performance TFTs. The IGZO precursor was coated on a heavily-doped silicon wafer covered with thermally grown silicon dioxide by spin coating followed by IPL annealing combined with deep-UV and NIR drying. The Ag source/drain (S/D) electrodes were screen-printed on the IPL-annealed IGZO layer and sintered by intense pulsed light while varying the irradiation energy.

## Results and discussion

### Annealing of IGZO semiconductor by thermal and IPL processes

**Figure 1:**
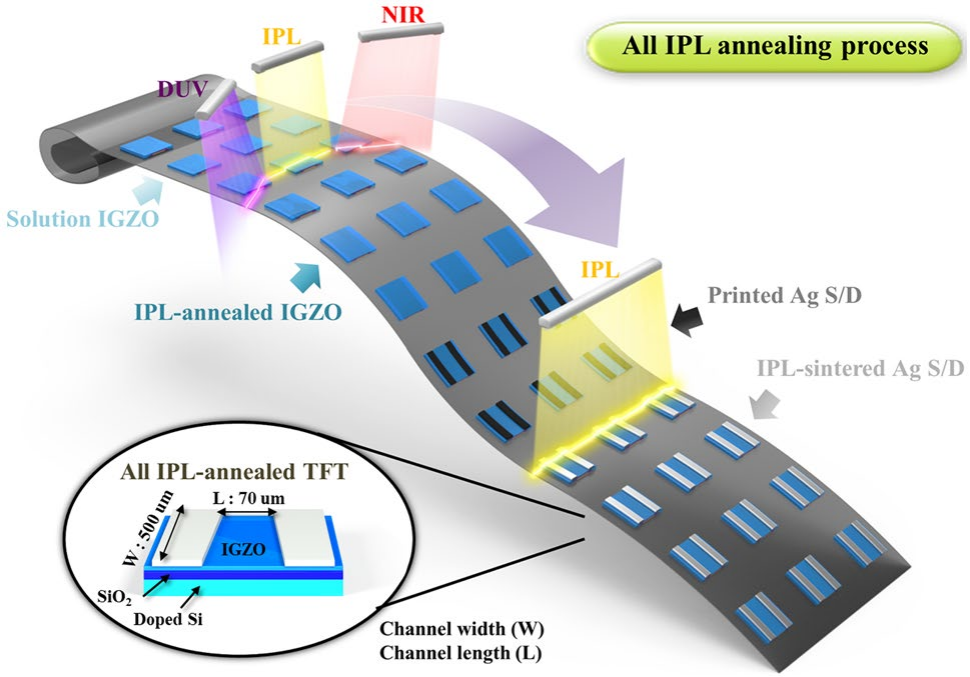
The schematic of all IPL annealing process of multi layers composed of IGZO semiconductor and Ag electrode (S/D). These processes are simultaneously progressed. The inset image is bottom gate TFT structure. (IGZO annealing condition: DUV with intensity of 60 mW/cm^2^, NIR with intensity of 3 W/cm^2^ was irradiated for drying and intense pulsed light with energy range from 70 to 130 J/cm^2^ was used for annealing; Ag sintering: intense pulsed light with energy range from 50 to 110 J/cm^2^ was applied for sintering).

In order to compare the effects of the annealing method of IGZO, the spin-coated IGZO precursor layer was annealed through thermal and IPL methods on prepared p++ Si/SiO_2_ substrates. As shown in Fig. 1, irradiation with intense pulsed white light, NIR, and DUV was conducted to dry and anneal the IGZO thin film semiconductor at room temperature under ambient conditions. The NIR and DUV irradiation, crucial for the drying process, were applied concurrently for 1 min. The NIR intensity (wavelength: 800–1,500 nm) was fixed at 3 W/cm^2^ and the intensity of the DUV (wavelength: 180–280 nm) was fixed at 60 mW/cm^2^, following our previous research^17^. This simultaneous application of NIR and DUV irradiation was a strategic choice: while NIR efficiently dries the coated IGZO precursor, the addition of DUV irradiation activates the bonding between Gallium (Ga) and Oxygen (O). This combined effect not only enhances the drying process but also plays a vital role in preventing the excessive generation of carriers in the IGZO layer, a common occurrence during IPL annealing due to oxygen separation. By maintaining high mobility and preventing the decrease in the on/off ratio, this dual irradiation approach optimizes the electrical properties of the IGZO thin films. This was followed by the IPL annealing process. To anneal the IGZO thin film semiconductor, the IPL energy was controlled from 70 to 130 J/cm^2^ with a total IPL annealing process time of 250 ms (ms), while constant temperature conditions were employed for 1 h during thermal annealing.

The Ag electrode was thermally evaporated on the annealed IGZO thin films to fabricate a bottom-gate TFT (inset image of Fig. 1, channel length: 70 µm, channel width: 500 µm). The TFT performance was calculated using the I (I_DS_)–V (V_G_) curve. The saturation mobility (µ_sat_) was obtained from the following equation.

1 $$\mu_{sat} = \frac{2L}{{W \cdot C_{i} }} \cdot \left( {\frac{{\partial \sqrt {I_{DS} } }}{{\partial V_{G} }}} \right)^{2}$$

**Figure 2:**
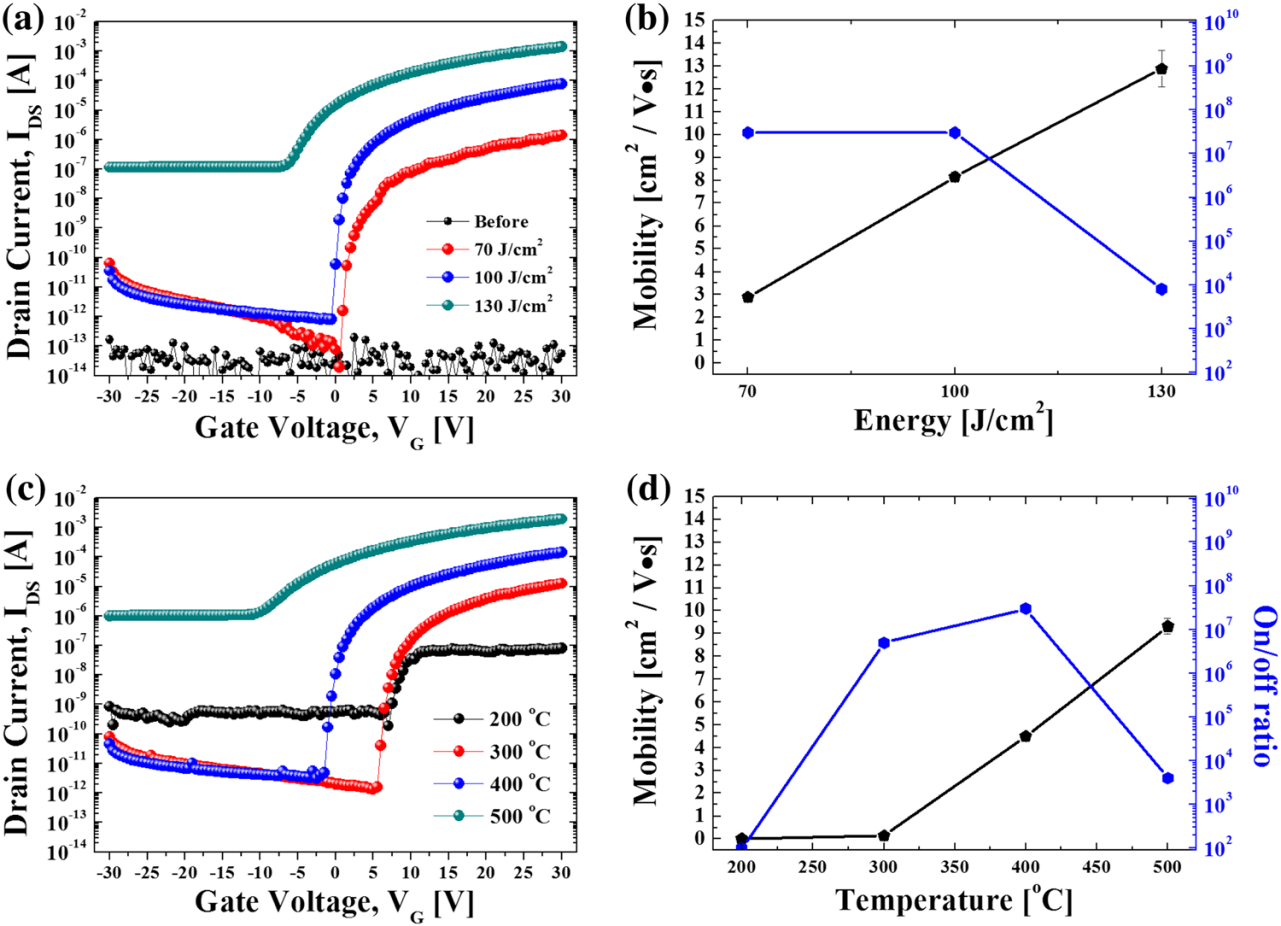
The electrical transfer properties (**a**) and the mobility and on/off ratio (**b**) of IPL-annealed IGZO and thermally evaporated Ag electrode based TFT (energy range: 70–130 J/cm^2^, pulse duration: 20 ms, pulse interval: 30 ms, 5 pulses, DUV, intensity 60 mW/cm^2^, NIR intensity: 3 W/cm^2^). The electrical transfer properties (**c**) and the mobility and on/off ratio (**d**) of thermal-annealed IGZO and thermally evaporated Ag electrode based TFT. (temperature from 200 to 500 degree).

Here, C_i_, W, and L denote the gate capacitance, channel width, and length, respectively. As shown in Fig. 2a, with increased IPL energy, TFT switching characteristics are clearly realized. Nevertheless, at an excessively high energy level of 130 J/cm^2^ IPL, the on/off ratio deteriorates due to an elevated off-current level. This is attributed to the formation of a conductive thin film with a mobility exceeding 10 cm^2^/V·s, echoing findings from our previous work involving a deposited Al electrode^17^. As shown in Fig. 2b, the IGZO thin film annealed with 100 J/cm^2^ IPL energy shows the best switching characteristics such as mobility and on/off ratio. In the case of the thermally annealed IGZO in Fig. 2c, different TFT characteristics were observed according to the annealing temperature. At an annealing temperature of 200–300 °C, low switching characteristics and high threshold voltage were observed. At 500 °C annealing, the semiconductor characteristics of the TFT were weakened due to excessive annealing conditions as large amounts of oxygen vacancies were generated in the IGZO thin film due to excessive heating, as reported in a previous report^17^. This shift in the threshold voltage, particularly observed in Fig. 2c compared to the near-zero threshold voltage in Fig. 2a, can be attributed to the different annealing temperatures and methods. The lower annealing temperatures (200–300 °C) in Fig. 2c resulted in reduced crystallinity in the IGZO layer, leading to decreased carrier mobility and an increased threshold voltage of around 5 V. In contrast, Fig. 2a showcases the application of DUV, NIR, and IPL irradiation, enhancing the crystallinity in the IGZO layer and bringing the threshold voltage closer to 0 V, indicative of more efficient charge carrier mobility at lower gate voltages. Comparing the IPL and thermal annealing cases in Fig. 2b and 2d, it was clearly shown that the IPL-annealed IGZO thin films showed better mobility and switching characteristics than those of thermally annealed IGZO. This is because in thermal annealing, IGZO is exposed to air during the thermal annealing in this work, which degrades the performance of IGZO^37^. In the case of IPL annealing, owing to its instant process time, the purity of the thin film can be maintained, thereby improving performance.

### Sintering of Ag source/drain(S/D) electrodes on IPL-annealed IGZO

**Figure 3:**
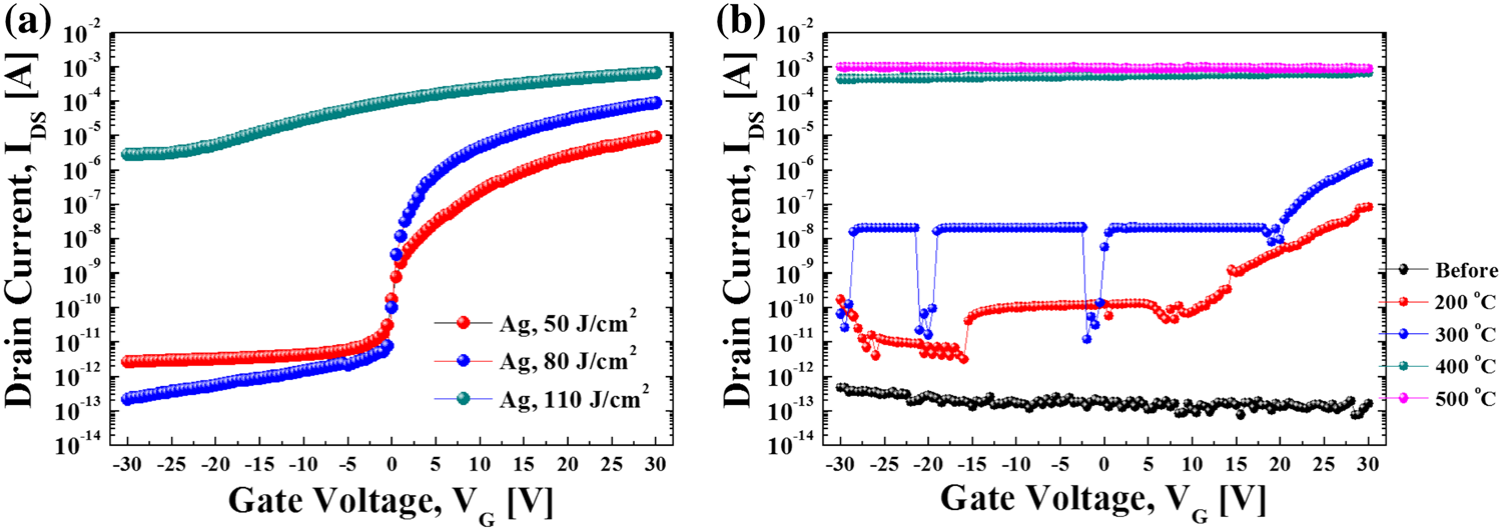
The electrical transfer properties of IPL-annealed IGZO and printed Ag electrode based TFT. (**a**) IPL-sintered Ag electrode based TFT and (**b**) thermal-sintered Ag electrode based TFT on optimally annealed IGZO with IPL energy of 100 J/cm^2^.

The Ag S/D electrodes were screen-printed using Ag nano ink and sintered on the optimally annealed IGZO with an energy of 100 J/cm^2^. As shown in Fig. 3a, when low IPL energy of 50 J/cm^2^ was irradiated on the printed Ag ink, the current switching and slope were not constant but increased in the on-state. Meanwhile, when the sintering energy was increased to 80 J/cm^2^, the TFT characteristics were stabilized in the on-state. In the case of 110 J/cm^2^ IPL energy, regarding the slope of the I–V curve, the TFT showed electrical conductive characteristics. On the other hand, the thermally sintered Ag electrode-based TFT did not show TFT characteristics at all under any sintering temperature conditions (see Fig. 3b). At a relatively low sintering temperature of 300℃, switching characteristics with low on/off ratios of about 10^2^ were observed. However, in all other conditions, only conductive characteristics were observed. Table 1 shows the TFT characteristics according to the electrode formation method. The IPL-annealed TFT (7.96 cm^2^/V s) showed similar characteristics to those of the conventional deposited electrode-based TFT (8.15 cm^2^/V s). Although the performance was slightly lower than that of the deposited electrode, IPL-annealed TFT can be considered to show excellent characteristics considering its simple solution process which does not require a sophisticated chamber system and can be conducted under ambient process conditions. Note that the full TFT fabrication consisting of source/drain electrodes as well as the IGZO layer using IPL annealing under ambient conditions is firstly reported here, although there have been previous reports about separate IGZO or S/D electrodes fabricated using IPL irradiation^17,37^. Furthermore, it has been previously reported that the characteristics of TFTs with printed and thermally sintered Ag ink electrodes were not good enough due to the reaction between Ag ink and IGZO and diffusion of Ag into the IGZO layer resulting from the continuously applied heat^34,35^. However, this phenomenon could be solved through the IPL sintering method owing to its instant sintering time.

**Table 1:** The performance of TFT by annealing method and S/D electrode deposition method.

Number	IGZO annealing method	Ag S/D electrode Deposition method	Mobility (on/off ratio)
1	IPL annealing	Thermal evaporation	8.15 cm^2^/V s (4 × 10^7^)
2	Thermal annealing	Thermal evaporation	4.5 cm^2^/V s (7 × 10^7^)
3	IPL annealing	IPL sintering	7.96 cm^2^/V s (3 × 10^7^)
4	IPL annealing	Thermal sintering	X (conductor)

### Characteristics of all-printed, IPL-annealed Ag electrode and IGZO layer

**Figure 4:**
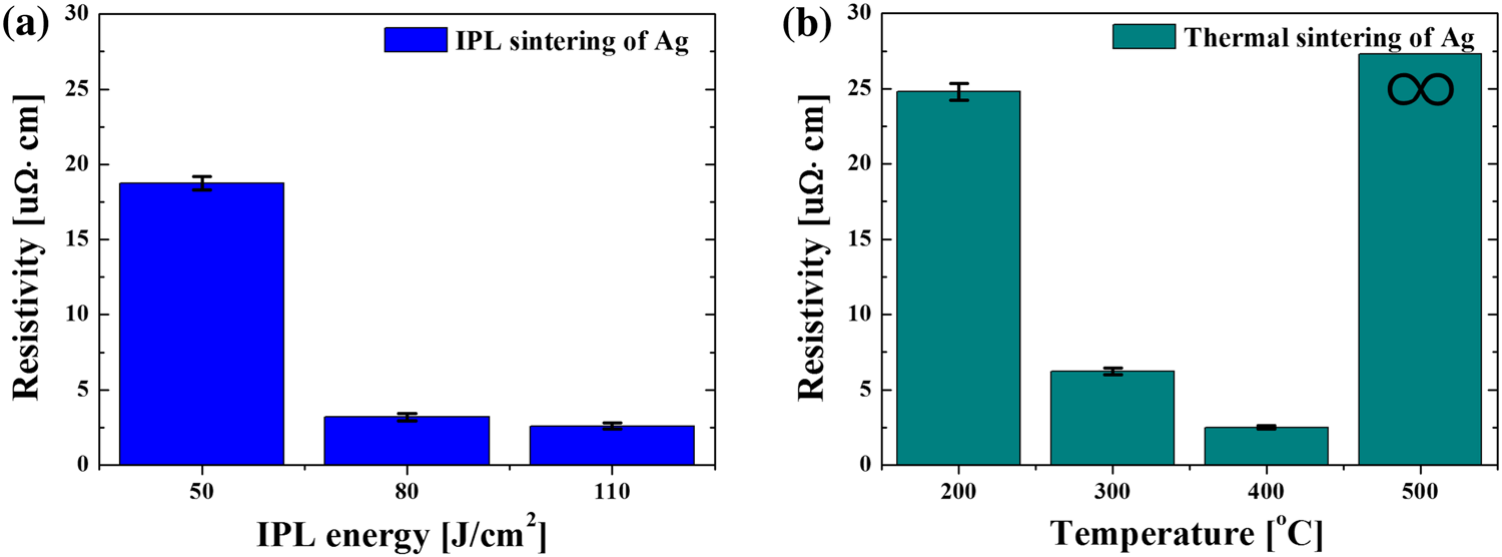
The electrical resistivity of Ag electrode line sintered by IPL (**a**) and thermal (**b**) on IPL-annealed IGZO.

**Figure 5:**
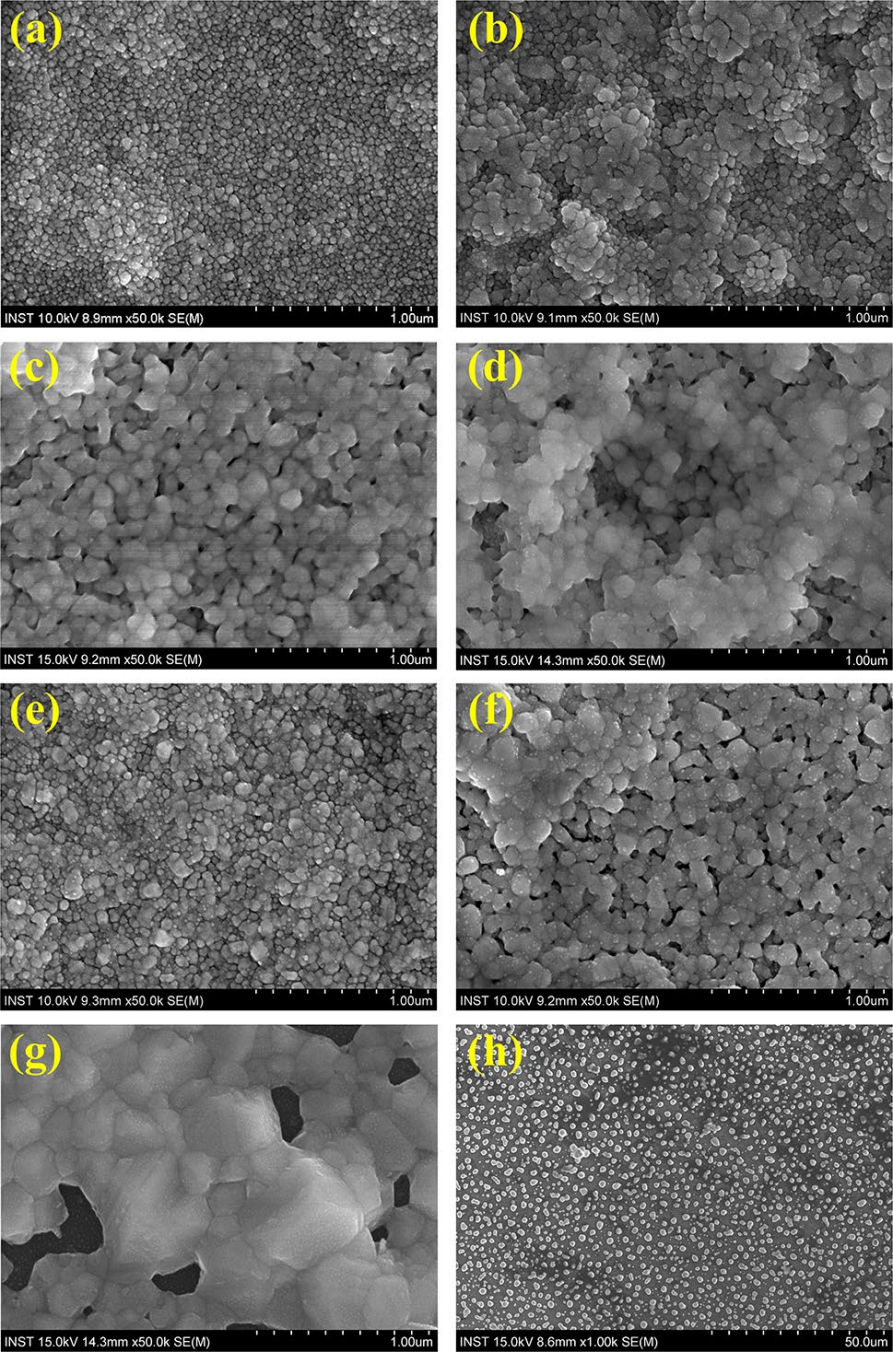
The microstructure of sintered Ag electrode on IGZO. (**a**) Before sintering. The IPL-sintered Ag electrode by energy of (**b**) 50 J/cm^2^, (**c**) 80 J/cm^2^ and (**d**) 110 J/cm^2^. The thermal-sintered Ag electrode by temperature of (**e**) 200 °C, (**f**) 300 °C, (**g**) 400 °C and (**h**) 500 °C.

The specific electrical resistance of the Ag electrode on IPL-annealed IGZO was evaluated with respect to the IPL sintering conditions. It has been reported that the degree of sintering of metal ink could affect its specific electrical resistance^32,33,40,43^ and the TFT characteristics varied depending on the specific resistance of the S/D electrode^27–29^. In this work, an Ag line with a length of 500 µm and width of 150 µm was printed and sintered on IPL-annealed IGZO. The thickness of the sintered Ag line differed according to the IPL sintering energy. Figure 4a shows the resistivity of the Ag line sintered under various IPL conditions. A low resistivity of 3.2 $\mathrm{\mu \Omega }$·cm was achieved in the case of IPL energies higher than 80 J/cm^2^ and it was saturated. The lower the resistivity of the Ag electrode, the better the switching characteristics of the TFT (see Fig. 3a). However, the IPL-sintered Ag electrode with an excessive IPL energy of 110 J/cm^2^ was damaged and showed no switching characteristics. Meanwhile, the specific resistance of the thermally sintered Ag electrode was the lowest at 400 °C and increased again in the case of sintering temperatures higher than 400 °C. It is noteworthy that although the Ag electrode was well sintered with a low resistivity of 2.5 uΩ cm at a thermal sintering temperature of 400 °C, the TFT showed no switching characteristics but conductive characteristics (see Fig. 3b). Figure 5 shows the microstructure of the Ag nanoparticles in the ink with respect to the sintering conditions. Before sintering, the Ag nanoparticles in the ink are surrounded by the binder and additives, thus contact among the nanoparticles was not formed (Fig. 3a). Generally, through the evaporation of the additives such as organic binder added to ink, the electrode structure evolves to a conductive pattern through densification between nanoparticles^32,33^. In the case of IPL sintering, the densest microstructure was observed at IPL energy irradiation at 80 and 110 J/cm^2^ (Fig. 5c and d), along with the lowest resistivity (Fig. 4a). In the case of the thermally sintered Ag electrode, the surrounding binder was removed and the particles were agglomerated in the 400 °C thermal sintering case (Fig. 5g). In the case of 500 °C, the Ag electrode structure was damaged and changed to an island structure, as shown in Fig. 5h, which made its specific resistance increase again (see Fig. 4b), similar to a previous report^34^. However, again, in spite of the well thermally sintered Ag electrode, the TFT characteristics were all converted to conductors rather than semiconductors (see Fig. 3b). This suggests that not only problems related to the sintering of the Ag electrode itself, but also the interface between the IGZO semiconductor and the Ag electrode and IGZO channel should be considered.

**Figure 6:**
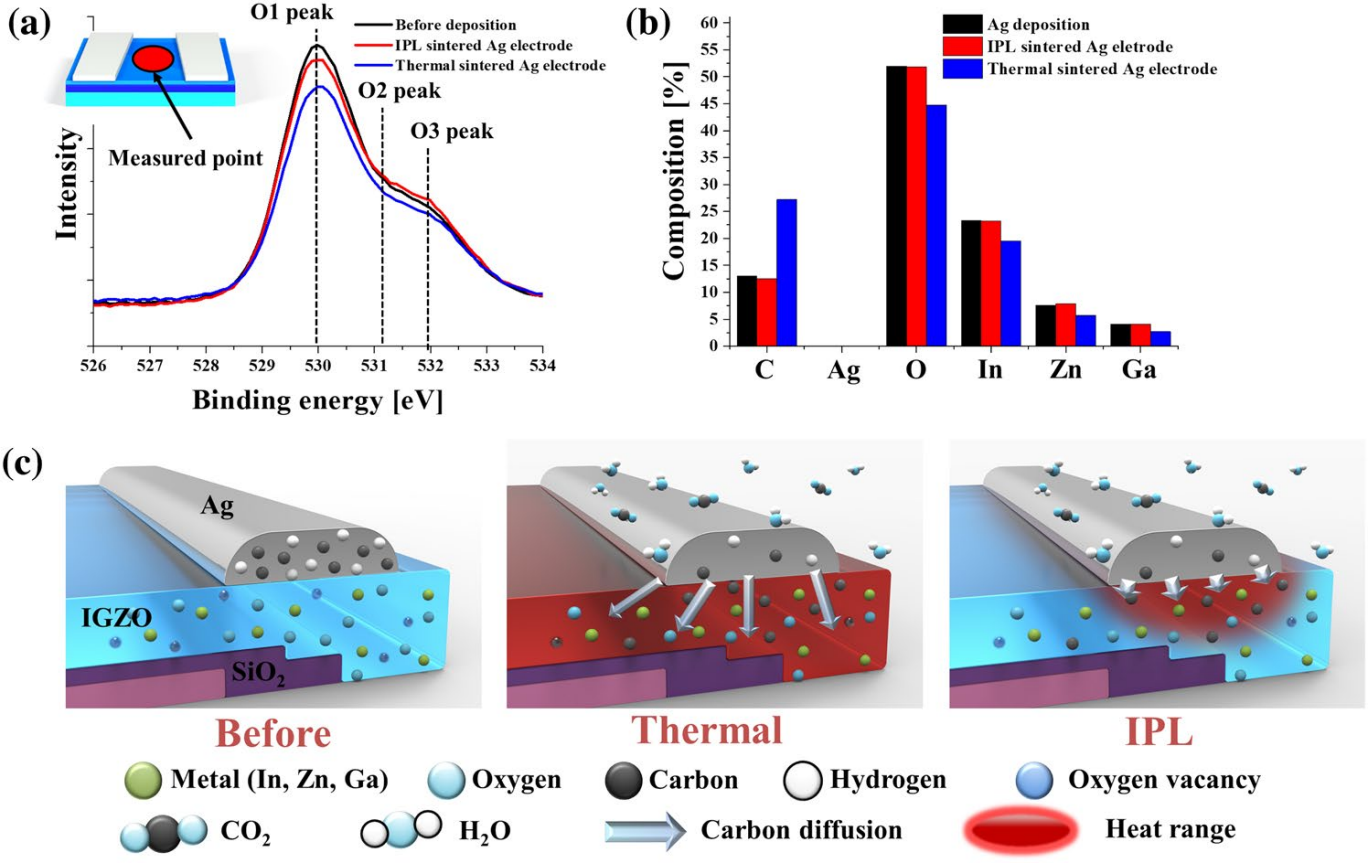
The chemical change of IGZO channel depending on sintering method of Ag electrode. (**a**) XPS O1s peak pattern for metal-oxide bond of IPL-annealed IGZO by electrode sintering method. (**b**) The chemical composition of IGZO channel depending on Ag electrode formation method. (**c**) schematic of reaction of organic materials on Ag ink and carbon diffusion by heat transfer according to sintering methods. (Ag electrode sintering condition: 80 J/cm^2^ for IPL, 300 °C for thermal).

In order to investigate the IGZO channel according to the sintering method, the chemical bonds and components in the IGZO channel formed between the S/D electrodes were analyzed through X-ray photoelectron spectroscopy (XPS), as shown in Fig. 6a and b. The range of the beam used for XPS analysis is an ellipse with a diameter of 450 µm. Thus, channels longer than 500 µm between two Ag lines were prepared followed by sintering and were analyzed. The O1s peaks in the IGZO layer occurs mainly at 530 eV (O1 peak), 531.2 eV (O2 peak), and 532 eV (O3 peak). The O1 peak is attributed to metal-oxide bonding (M–O), the O2 peak located at 531.2 eV reflects the oxygen vacancies (Vo), and the O3 peak located at 532 eV corresponds to the existence of weakly bound oxygen species on the film surface such as -OH^46^. Among them, the O1s peak shows the most important bonds for the semiconductor properties of the IGZO composition. Figure 6a shows that the O1s peak (metal-oxide bonding (M–O)) in the IGZO channel changed according to the sintering method. Also, the combination of materials composed of the IGZO channel became different according to the sintering method, as shown in Fig. 6b. In the IGZO channel region between Ag S/D, the Ag content was not detected completely in all cases, as shown in Fig. 6b, which is good as Ag ions diffused into the IGZO layer can degrade the TFT performance^35^. The amounts of In, Ga, Zn, and O, which determine the performance of the IGZO semiconductor, were not significantly different according to the Ag sintering method. Meanwhile, the carbon (C) content on IGZO with the deposited or IPL sintered Ag lines was significantly lower than that of IGZO with the thermally sintered Ag lines. In the case of IPL sintering, the carbon content was around 10% and it increased by a factor of two in the case of thermal sintering. This reveals that the channel of the TFT might be damaged by the carbon diffused during the thermal sintering process. The diffused carbon might originate from organic substances such as the binder and solvent in the Ag ink, which decompose into carbon. As the annealing process is progressed in the thermal sintering process, the carbon may be released into the air adjacent to IGZO and may diffuse into IGZO due to the continuous heat in the thermal sintering process, as depicted in Fig. 6c. On the other hand, in the case of the IPL-sintered electrode, the diffusion of carbon can be minimized due to its instant sintering.

**Figure 7:**
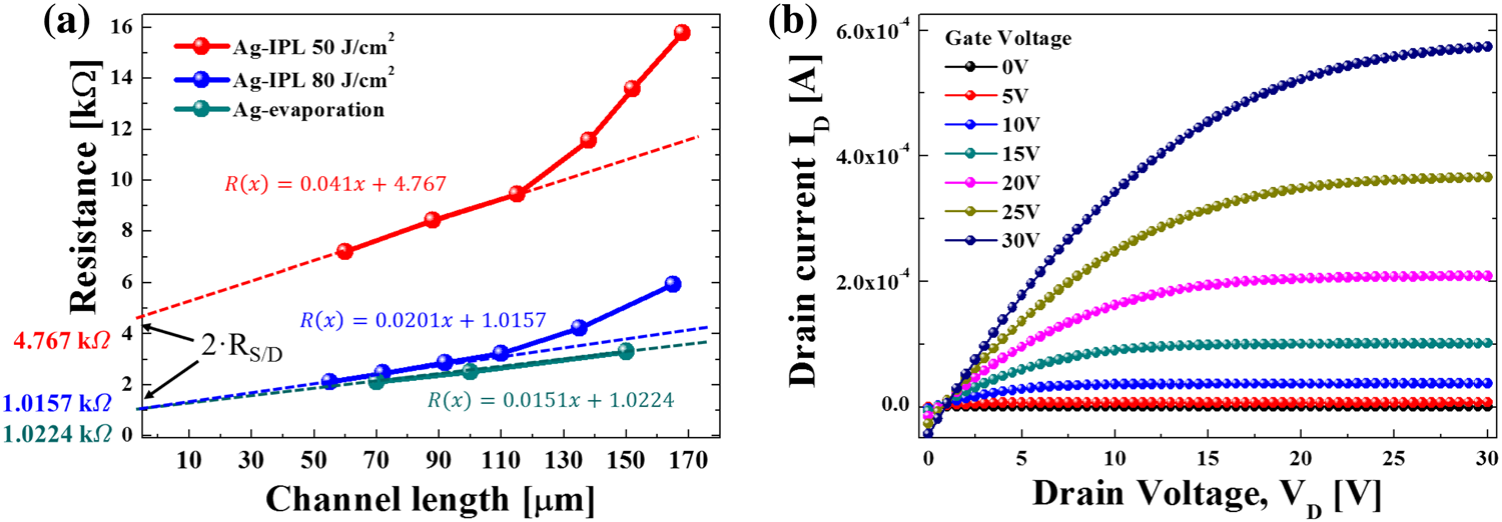
(**a**) The contact resistance of interface with IPL-sintered Ag electrode and IPL-annealed IGZO by Ag electrode deposition method. (**b**) The output property of optimally all IPL annealed TFT.

To evaluate the electrical properties between the interface of IGZO and Ag, the contact resistance was calculated from TFT patterns fabricated at various channel lengths, as shown in Fig. 7. The transmission line method (TLM) was adopted to evaluate the contact resistance of the interface of IGZO and Ag^47^. By defining the total TFT On resistance (R_T_) as R_T_ = V_DS_/I_DS_, R_T_ can be expressed as follows:

2 $$R_{T} = \frac{{V_{DS} }}{{I_{DS} }} = r_{ch} \cdot L + 2R_{S/D}$$

where 2R_S/D_ is the total source and drain contact resistance with IGZO and r_ch_ is the channel resistance per channel length, which is defined as the slope of R_T_ versus the source-to-drain distance. The measured total resistance, R_T_, was plotted and R_S/D_ was calculated based on the slope and intercept, as shown in Fig. 7a, according to the IPL sintering condition. Comparing the contact resistance results for the IPL sintering condition of Ag, the lowest contact resistance of 508 Ω was found at 80 J/cm^2^. In the case of 50 J/cm^2^, the contact resistance was higher because the heat converted by light was not sufficient and the binder surrounding the Ag nanoparticles could not be removed (Fig. 5b). In addition, due to such a high contact resistance, the TFT characteristics were not good enough, as shown in Fig. 3a. Please note that the IGZO annealing conditions were the same in all cases in this work (100 J/cm^2^ of IPL energy for annealing and DUV with an intensity of 60 mW/cm^2^, NIR with an intensity of 3 W/cm^2^ for drying). The optimal contact resistance of the Ag electrode sintered with an IPL irradiation energy of 80 J/cm^2^ with IGZO was found to be about 500 Ω, which is almost the same as that of a deposited Ag electrode-based TFT (see Fig. 7a). Figure 7b shows the output properties of the printed Ag/IGZO TFT under optimum conditions (80 J/cm^2^ IPL energy for the Ag electrode and 100 J/cm^2^ IPL energy combined with DUV drying with an intensity of 60 mW/cm^2^ and NIR with an intensity of 3 W/cm^2^ for the IGZO layer) with varying drain voltages. Under gate voltage conditions, it could be seen that the current values of the TFTs reach a stable saturation state and at gate voltages above 20 V, the current level increased to the 10^–4^ A level, as shown in Fig. 7b. As the voltage applied to the TFT increases, the drain current increases, which is consistent with the transfer property of the optimally IPL-annealed TFT (Fig. 3a). From these results, it could be concluded that TFTs with excellent performance can be realized with the optimal IPL sintering and annealing conditions of the Ag electrode and IGZO layers, respectively.

**Figure 8:**
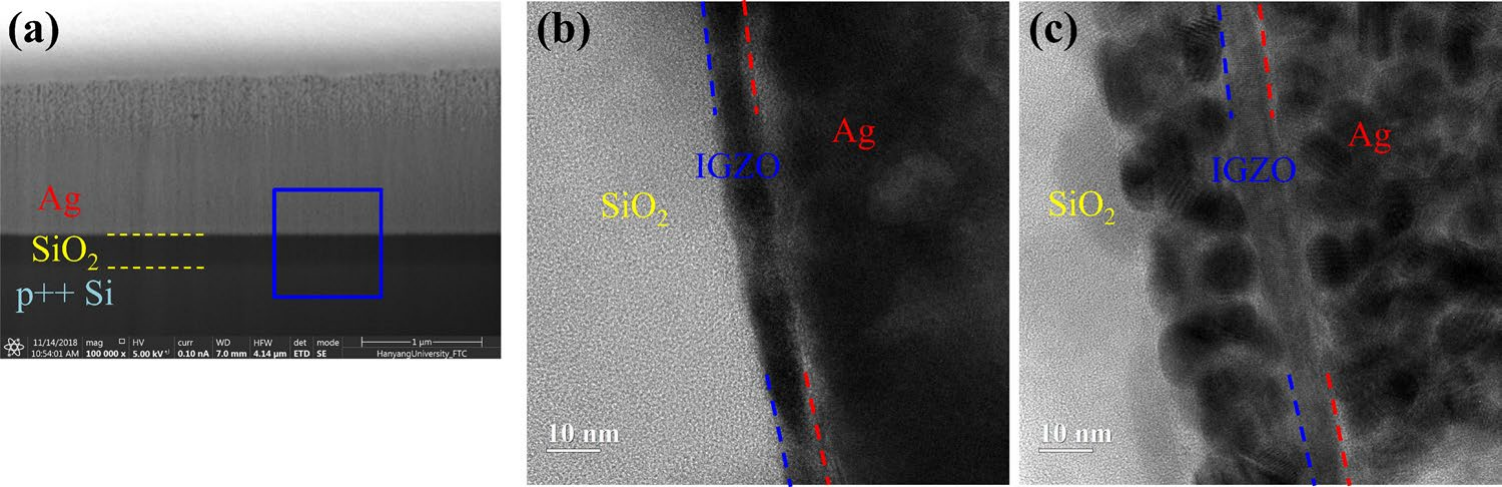
The cross sectional stacked structure images of all IPL annealed TFT (**a**), TEM image of IPL sintered Ag electrode based TFT (**b**) and thermally sintered Ag electrode based TFT (**c**). The Ag-IGZO-SiO_2_ stacked structure in blue square was analyzed. (Ag electrode sintering condition: 80 J/cm^2^ for IPL, 300 °C for thermal).

For deeper understanding of the interface of the all-printed optimally IPL-annealed TFT, cross-sectional analysis was performed, as shown in Fig. 8. It has been reported that, in the IPL sintering process, due to the heat generated, damage to the film or delamination on the substrate can occur^40,44^. However, it is noteworthy that the well-formed laminated interfacial structure showed not only the well sintered Ag electrode itself (Fig. 5c), but also a dense and well sintered cross-section and interface between the Ag layer and IGZO layers in the thickness direction could be formed, as shown in Fig. 8a. In the interface of IGZO and Ag, the three layers of SiO_2_, IGZO, and Ag were stacked well side by side, as shown in Fig. 8b. This phenomenon can also be confirmed in the Time-of-Flight Secondary Ion Mass Spectrometry results in Figure S2 of supplementary information. In this interface, metal materials often infiltrate the semiconductor layer or penetrate the semiconductor layer into SiO_2_^48,49^. Again, this might be because that chemical changes by diffusion and physical damage can be minimized through the instant IPL annealing process. In order to compare the performance and process efficiency of all-printed, IPL-annealed TFT, the characteristics and processing methods of recently published research on solution-processed metal oxide semiconductor and metal electrode-based TFTs are summarized and compared in Table 2. For comparison under the same conditions, TFTs with metal oxide and metal electrode stacked on doped Si/SiO_2_ were chosen while the cases with a stacked structure consisting of other dielectrics were excluded. In recent decades, many researchers have focused on improving the performance of metal oxide through the improvement of semiconductor materials, annealing method, and structure improvement of TFTs^9,17,42,50–58^. The development of In and Zn-based oxide semiconductors was mainly achieved and most of them used aluminum as an electrode deposition material^17,42,50–58^. The mobility of TFTs showed performances of about 10 cm^2^/V s. However, as mentioned above, the focus in previous research was only focused on improving the semiconductor layer. Research of the electrode and semiconductor annealing has not been conducted yet. Specific studies used multi layers of semiconductor and a S/D electrode layer, but the mobility performance of TFTs was less than 5 cm^2^/V·s, which is much lower compared to the deposition electrode^35,36,59–67^. Contrary to the metal electrode, when graphene and ACO (aluminum-doped cadmium oxide) were used, the TFT performance was greatly improved^68,69^. Both materials could exhibit high TFT properties, forming low contact resistance with the oxide semiconductor. However, they had to be fabricated using a high temperature above 250℃ and a long thermal annealing process of 1–2 h^68,69^. The total process time would increase significantly to several hours considering all stacked layers in TFT structures. On the other hand, again, the all-printed and IPL annealing process for TFT fabrication proposed in this work can print and anneal multi-stacked layers for TFTs in several minutes, without requiring a repetitive heat chamber or an inert gas system.

**Table 2:** Recently announced performances of metal oxide based or printed electrode based TFTs according to annealing method and electrode deposition method.

#	Semiconductor materials	Annealing method (temperature, time)	Electrode materials	Deposition method	Mobility (on/off ratio)	Year (ref)
1	IGZO	Thermal (350, 2 h)	MoNb alloy	Magnetron sputtering	6.2 (10^7^)	2016 (50)
2	IGZO	Thermal (300, 20 min)	Al	Thermal evaporation	8.2 (10^7^)	2016 (51)
3	IGZO/SWCNT	DUV (200, –)	Al	Thermal evaporation	3.54 (–)	2019 (52)
4	In_2_O_3_	UV (150, 15 min)	Al	Thermal evaporation	34.44 ()	2016 (53)
5	In_2_O_3_	Light (200–2000 nm, RT)	Al	Thermal evaporation	10.3 (10^6^)	2016 (42)
6	IGZO	White light (RT, 1 s)	Ti/Au	sputtering	0.001 (10^5^)	2015 (54)
7	IGZO	IPL + DUV + NIR (RT, 1 min)	Al	DC sputtering	7.7 (10^6^)	2019 (17)
8	In_2_O_3_	Laser-KrF excimer (RT, –)	Al	Thermalevaporation	13 (10^6^)	2017 (55)
9	IGZO	Plasma (250, –)	–	–	3.8 (10^6^)	2018 (56)
10	ZTO	Thermal (500, –)	ITO/Au	–	7.8 (10^8^)	2016 (57)
11	ZnO/SnO_2_	Thermal (500, 2 h)	Al	Thermal evaporation	20.7 (–)	2016 (58)
12	IGZO/In_2_O_3_	DUV (150, 2 h, N2)	IZO	–	2.3/3.6 (10^4^)	2012 (9)
13	IGZO	Thermal (400, 2 h)	Ag	Printing/thermal (80, 2 h)	2.6 (–)	2017 (35)
14	In_2_O_3_-PEI	Thermal (300, 30 min)	Ag	Printing/thermal (150, 30 min)	3.0 (–)	2018 (36)
15	ZTO	Thermal (600, 1 h)	Ag	Printing/thermal (150, 1 h/vaccum)	2.3 (10^6^)	2011 (59)
16	ZTO	Thermal (500, 1 h)	Cu	Printing/thermal (250, 10 min /N_2_)	2.6 (10^5^)	2015 (60)
17	IGZO	Thermal (500, 1 h)	Ag	Printing/thermal (130, 30 min)	0.2 (10^6^)	2009 (61)
18	ZnO	Thermal (400, 30 min)	Ag/ITO	Printing/thermal (400, 30 min)	0.53 (10^4^)	2016 (62)
19	IGZO	Thermal (300, 45 min, humidity)	Graphene	Printing/thermal (300, –)	6 (10^5^)	2016 (68)
20	InO_x_	Thermal (250, 2 h)	ACO	Printing/thermal (250, 160 min)	19 (–)	2017 (69)
21	IGZO	Thermal (400, 1 h)	Cr/Au, Ga/Au, In/Au	DC sputtering	5.67, 11.40, 12.67 (–)	2023 (63)
22	a-IGZO/IZO	(–)	Mo/Pt	RF magnetron sputtering	17.22 (–)	2021 (64)
23	a-IGZO	Thermal (300, 1 h)	Al	Thermal evaporation	12.9 (0)	2020 (65)
24	a-IGZO	(–)	ITO	RF sputtering (400, 2 h)	52.48 (10^9^)	2022 (66)
25	a-IGZO	(–)	Al	Thermal evaporation	5.8 (1.3 × 10^9^)	2024 (67)

The metal oxide based semiconductors were classified by annealing method, temperature and time, and the electrode was summarized by deposition method.

A part not introduced in the research was marked with (–). In the case of using a gas atmosphere, it was additionally indicated.

## Conclusion

In this study, an all printing and IPL annealing process for TFTs was carried out using solution processed IGZO and a printed Ag electrode without a thermal process and deposition method. All-printed and IPL-annealed TFTs could be realized with a high mobility of 7.96 cm^2^/V·s and on/off ratio of 10^7^, which is comparable to those of a deposited electrode-based TFT. Considering the thermal sintering of an Ag electrode on IGZO, it was concluded that a thermal-sintered Ag electrode-based TFT could not be developed due to the diffusion of carbon during the sintering process. The IPL annealing method was found to be effective not only for annealing IGZO but also for sintering of the electrode on a TFT structure. Also, the all IPL annealing process was able to form structurally, chemically, and electrically stable electrodes and IGZO as well as their interface, thus enabling the implementation of high performance TFTs. Through the all IPL printing and annealing process, it was possible to fabricate all-printed inorganic TFTs at a high production speed under ambient conditions at room temperature instantly. The proposed process is expected to open a new path in the IGZO TFT field.

## Methods and materials

### Fabrication of TFT materials (IGZO solution and Ag paste)

The IGZO precursor solutions were prepared by mixing zinc nitrate hydrate, indium nitrate(III) hydrate, and gallium nitrate(III) hydrate in 2-methoxyethanol and all materials associated with IGZO were purchased from Sigma Aldrich Co. The total concentration of the solution was 0.12 M and the molar ratio was adjusted to 6.5(In): 1.5(Ga): 2(Zn). This solution was vigorously stirred (600 rpm) over 12 h. Meanwhile, a heavily p-type doped silicon wafer covered with 300 nm thick, thermally grown SiO_2_ was sonicated in acetone and isopropyl alcohol for 5 min. In order to remove the residual solvent on the wafer, N_2_ gas was blown onto the wafer using a blower system. The substrates were treated with ultraviolet-ozone for 5 min. To produce the high viscosity inks used in the screen-printing process, ethyl cellulose (Sigma Aldrich Co.) was added to commercial Ag ink (Silverjet DGP, ANP Co.) at a weight fraction of 1%. The ink was stirred and heated to a temperature of 75 °C for 2 h to reduce the solvent content and increase the Ag particle weight percent.

### IPL annealing of IGZO with DUV and NIR irradiation

An IGZO solution was coated using a spin-coater (SC-200, Nano-tech) at 3000 rpm for 30 s on a prepared p++ Si/SiO_2_ substrate. Irradiation with intense pulsed white light, NIR, and DUV was conducted to dry and anneal the IGZO thin film semiconductor at room temperature under ambient conditions (Fig. 1). The NIR and DUV were irradiated simultaneously to dry the coated IGZO precursor layer for 1 min. The NIR intensity (wavelength: 800–1500 nm, Adphos L40) was fixed at 3 W/cm^2^ and the intensity of the DUV (wavelength: 180–280 nm, Lumatec SUV-DC) was fixed at 60 mW/cm^2^. The intense pulsed light system consisted of a xenon flash lamp (PerkinElmer Co.), a power supply, capacitors, a pulse controller, and a water cooling system (myPET™, Semisysco Co.). The intense pulsed light from the xenon flash lamp has a broad wavelength range (380–950 nm) and the IPL energy was varied from 70 to 130 J/cm^2^ while the pulse duration time, gap time, and pulse number were fixed at 20 ms, 30 ms, and 5, respectively, following a previous work^43^. In comparison, the coated IGZO film was thermally annealed in the furnace for 1 h at 200, 300, 400, and 500 °C under atmospheric conditions.

### Fabrication of the Ag S/D electrode on IGZO for the bottom gate TFT structure

Ag ink was screen-printed on an IPL-annealed IGZO layer with a channel length of 70 µm between the S/D electrode and width of 500 µm (inset images of Fig. 1). The printed electrodes were sintered in two ways: IPL and furnace heating. Firstly, the Ag electrodes on IGZO were fabricated by thermal sintering in a furnace for 10 min at various temperatures ranging from 200 to 500 °C. Secondly, the printed Ag electrode was sintered at room temperature under ambient conditions through IPL. Before irradiating the intense pulsed light, the Ag electrode was dried for 1 min through NIR with an intensity of 3 W/cm^2^ and the Ag electrode was sintered by adjusting the IPL energy from 40 to 60 J/cm^2^ with a pulse duration time, gap time, and pulse number of 20 ms, 30 ms, and 5, respectively (Fig. 1). For comparison, 200 nm thick Ag S/D electrodes were deposited on the IPL-annealed IGZO surfaces by a thermal evaporator system.

### Characterization of TFTs according to the sintering conditions of the Ag electrode

To estimate the electrical properties of the IGZO-based TFTs, the current–voltage characteristics of the TFTs were measured using a parameter analyzer (4200-SCS, Keithley Instruments, Inc.) under ambient conditions in a dark box. The drain voltage was fixed at 30 V and the gate voltage was controlled from − 30 to 30 V. The TFT characteristics such as mobility and on/off ratio were calculated according to the fabrication method of the Ag electrode as well as the annealing conditions of IGZO. In order to evaluate the electrical resistivity of the sintered Ag electrode, the resistance of a line with a 500 µm length and 150 µm width was measured by a parameter analyzer. In addition, the thickness was measured. The microstructure of the sintered electrode was observed by a scanning electron microscope (SEM, S4800, Hitachi Co.). In order to analyze the chemical change of the IGZO channel according to the sintering conditions, component analysis was performed using XPS (K-alpha, Thermo Fisher Scientific Co.). The structural stability of the fabricated TFTs was estimated using a focus ion beam (FIB, Scios, FEI Co.) and transmission electron microscope (TEM, JEM 2100F, JEOL Co.).

### Supplementary Information

Supplementary Information.

## Data Availability

The datasets used and/or analyzed during the current study available from the corresponding author on reasonable request.
