# Supplementary material for: Intense pulsed light annealing of solution-based indium–gallium–zinc–oxide semiconductors with printed Ag source and drain electrodes for bottom gate thin film transistors
Source: Sci Rep. 2024 Jan 18;14:1566. doi: 10.1038/s41598-024-52096-2 (PMC10796356; doi:10.1038/s41598-024-52096-2)
Supplement: Supplementary file 1 — Supplementary Information. [file 41598_2024_52096_MOESM1_ESM.docx]

**Supplementary Information**

**Intense Pulsed Light Annealing of Solution-Based Indium-Gallium-Zinc-Oxide Semiconductors with Printed Ag Source & Drain Electrodes for Bottom Gate Thin Film Transistors**

Chang-Jin Moon^1^, Jong-Whi Park^1^, Yong-Rae Jang^1^ and Hak-Sung Kim^1,2*^

**^1^**Department of Mechanical Engineering, Hanyang University, 17 Haengdag-Dong, Seongdong-Gu, Seoul 133-791, South Korea

**^2^**Institute of Nano Science and Technology, Hanyang University, Seoul, 133-791 South Korea

* Corresponding author. Tel: +82-2-2220-2898

E-mail address: [kima@hanyang.ac.kr](mailto:kima@hanyang.ac.kr) (Hak-Sung Kim)

**I. AFM Analysis of Surface Roughness in Annealed Ag Electrodes**

The AFM data for Ag electrodes without any annealing treatment showed a surface roughness of 78.483 nm as shown figure S1(a). This measurement serves as a baseline for comparing the effects of the subsequent annealing processes. After thermal annealing process (400 $℃$), the surface roughness of the Ag electrodes was significantly reduced to 31.47 nm as shown figure S1(b). This reduction indicates the effectiveness of the thermal annealing process in smoothing the electrode surface. The AFM results for the Ag electrodes annealed with IPL irradiation process (80 J/cm^2^) showed the lowest surface roughness of 27.019 nm as shown figure S1(c). This reduction compared to the thermally annealed samples suggests the superior efficacy of IPL annealing in achieving a smoother electrode surface.

In conclusion, the AFM analyses provide vital insights into the surface morphologies of Ag electrodes induced by different annealing methods. The progressive decrease in surface roughness from the non-annealed to the IPL annealed one demonstrates the significant impact of annealing techniques on the micro-structure of the Ag electrodes. Notably, IPL annealing appears to be the most effective in reducing surface roughness, potentially influencing the electrical and mechanical properties of the electrodes. The detailed AFM data, alongside the SEM imagery in the main text, offers a comprehensive understanding of the surface modifications imparted by the annealing processes on Ag electrodes.


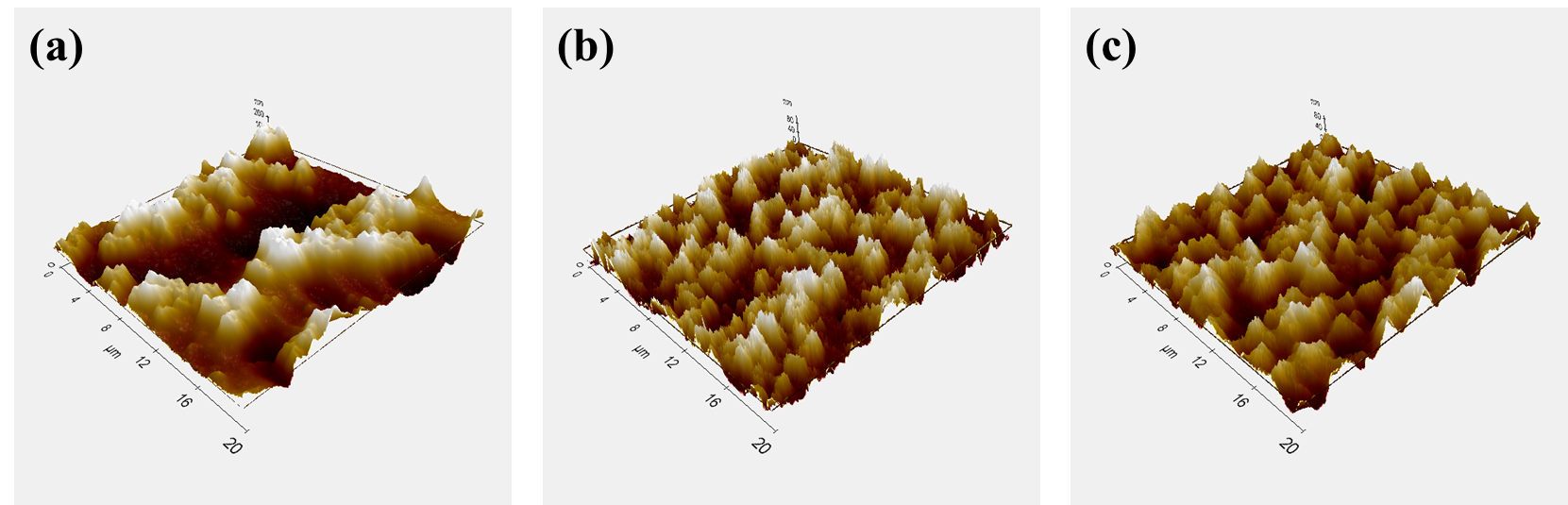


**Figure S1.** The AFM analysis data of Ag electrodes: **(a)** Before sintering, **(b)** Thermal annealing with 400 $℃$, **(c)** IPL annealing with 80 J/cm^2^.

**Ⅱ. ToF-SIMS Analysis of interface with Ag layer and IGZO layer**

To understand the interfacial interactions between Ag and IGZO layers, Time-of-Flight Secondary Ion Mass Spectrometry (ToF-SIMS) was employed. This analysis is important for assessing the elemental distribution, particularly focusing on carbon and silver, across different layers and formation methods of the Ag electrode. ToF-SIMS offers a depth profile, revealing how these elements are distributed and interact within the layers, providing crucial insights into the chemical and physical changes occurring during different annealing processes. Figure S2 displays the ToF-SIMS analysis results, illustrating the distribution of (a) carbon and (b) silver elements in the Ag and IGZO layers. The analysis was conducted for electrodes prepared through various methods, including thermal annealing at 300 ℃ and IPL annealing at 100 J/cm2, and the deposited Ag electrode. The data from Figure S2(a) indicates the presence and variation of carbon content across different layers and electrode formation methods. It was observed that thermal annealing resulted in a noticeable migration of carbon into the IGZO layer, a phenomenon less pronounced in the IPL annealed and deposited samples. This difference in carbon distribution was crucial, as it directly influences the electrical properties of the layers, especially regarding carrier mobility and conductivity. Similarly, Figure S2(b) showed the Ag element distribution. The depth profiling provided by ToF-SIMS was shown how Ag diffuses into the IGZO layer under different annealing conditions. The analysis indicates a higher degree of Ag diffusion in thermally annealed electrodes as compared to IPL annealed and deposited samples.


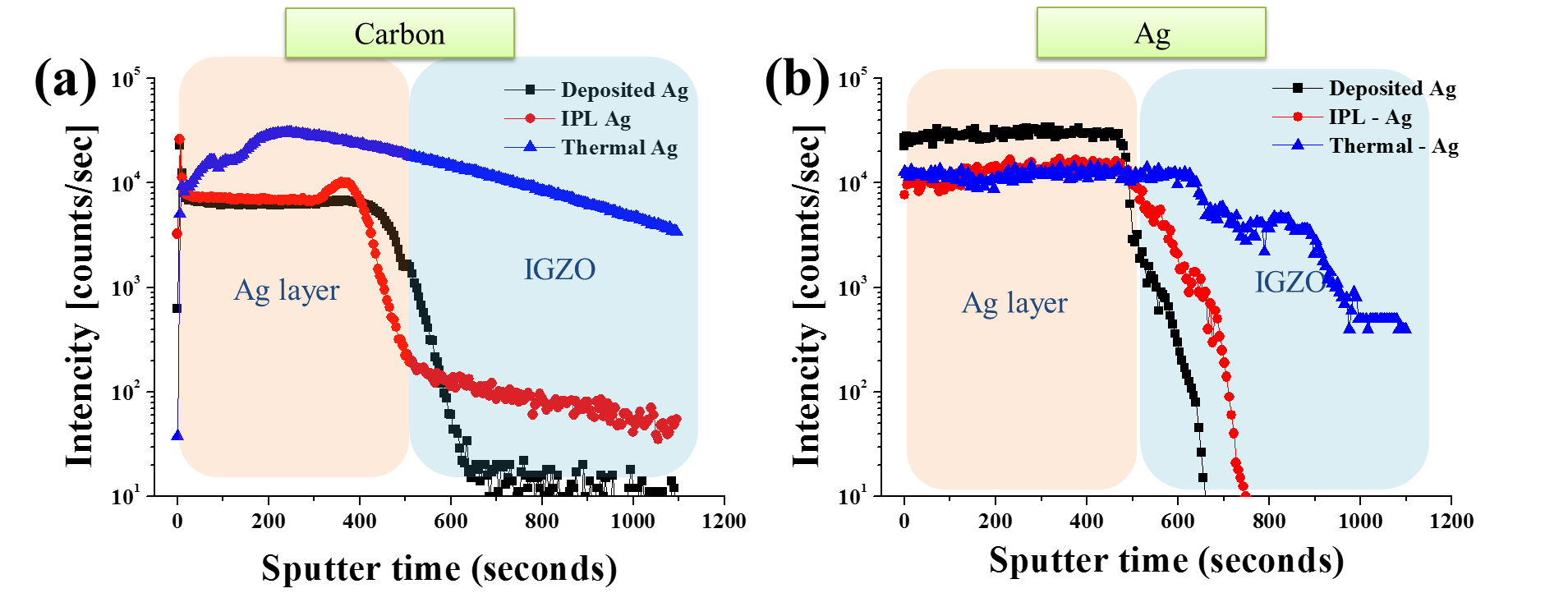


**Figure S2.** The amount of (a) carbon and (b) silver elements distributed in the Ag and IGZO layers by formation method of Ag electrode.
